# Altered serum metabolome is associated with disease activity and immune responses in rheumatoid arthritis

**DOI:** 10.1007/s10067-024-07201-1

**Published:** 2024-11-01

**Authors:** Xuanlin Cai, Jiayang Jin, Hua Ye, Xiaohong Xiang, Li Luo, Jing Li

**Affiliations:** 1 Department of Rheumatology and Immunology, First Affiliated Hospital of Xinjiang Medical University, Urumqi, 830011 Xinjiang China; 2 Department of Rheumatology and Immunology, Peking University People’s Hospital, Beijing, 100044 China; 3 Beijing Key Laboratory for Rheumatism Mechanism and Immune Diagnosis (BZ0135), Beijing, 100044 China

**Keywords:** Rheumatoid arthritis, Metabolomics, Disease activity, Immune responses

## Abstract

Rheumatoid arthritis (RA) is widespread globally, with the emergence of metabolites derived from both the host and microbes playing a pivotal role in its pathogenesis. This study aims to elucidate the relationships between serum metabolites and the immunological and clinical features of RA. Serum samples were collected from 35 RA patients and 37 healthy controls (HC). Metabolite profiling was performed using gas chromatography-mass spectrometry (GC/MS). Principal component analysis revealed a significant distinction between the RA and HC cohorts. Employing univariate statistical analysis, we identified 36 differential metabolites. Among these, 9 metabolites, including galactose and glucose, were found to be enriched, while the remaining metabolites, such as citric acid, fumaric acid, and inosine, were depleted in RA. These diverse metabolites encompassed various metabolic processes, including the biosynthesis of fatty acids, amino acids, and glucose. The enrichment of glucose and galactose in RA exhibited a substantial correlation with elevated IgG levels, as determined through correlation analysis. Conversely, the depletion of citric acid was correlated with elevated levels of C3 and CRP. Methionine, which also declined in RA patients, displayed a negative correlation with ESR. Furthermore, galactose and glucose exhibited significant positive correlations with naïve B cells, while the decreased eicosanoic acid level in RA was significantly associated with an increase in natural killer cells. Our findings suggest that the altered serum metabolite profile in RA is closely linked to disease severity and the dysregulated immune responses observed in RA patients.

**Table:** 

**Key Points**
• *Identified nine metabolites with upregulated expression and twenty-seven metabolites with downregulated expression.* • *Established a correlation between alterations in serum metabolite levels and inflammatory markers in RA patients.* • *Discovered a significant association between changes in serum metabolites and immune cell profiles in RA patients.*

**Supplementary Information:**

The online version contains supplementary material available at 10.1007/s10067-024-07201-1.

## Introduction

Rheumatoid arthritis (RA), an autoimmune disorder, is characterized by joint inflammation that, in severe cases, can lead to disability and irreversible joint damage. RA can also impact other organs [1]. The prevalence of RA is roughly 0.5–1% in the population, with females being 2–3 times as likely as males to develop the condition [2]. RA is a symmetrical, multi-joint disease, often marked by morning stiffness lasting more than 30 min and joint pain and swelling in the hands and feet [3, 4]. Extra-articular symptoms and comorbidities are prevalent in RA, contributing to increased severity and reduced survival. The chronic inflammatory state of RA can lead to the development of rheumatoid nodules, vasculitis, and involvement of cardiovascular, pulmonary, and other organs [5].

The pathogenesis of joint inflammation in RA is driven by complex interactions involving various innate immune cells, T cells, B cells, and other immune cells, as well as fibroblasts and osteoclasts [6, 7]. Dysregulated immune responses play a critical role in sustaining disease progression.

Metabolomics is a flourishing field of study that employs high-throughput analytical techniques to comprehensively investigate metabolites in living organisms [8]. Metabolites, which are endogenous products of the host, play essential roles in regulating apoptosis, cell signaling, energy production, and other vital cellular processes [9]. In clinical practice, metabolomics is primarily employed for identifying diagnostic biomarkers, analyzing potential new drug targets, exploring pathological mechanisms, and detecting therapeutic responses [10]. Metabolomics has gained increasing relevance in understanding autoimmune diseases due to its capacity to uncover functional mechanisms underlying different diseases. Studies have delved into the roles of glycolysis, the tricarboxylic acid (TCA) cycle, the pentose phosphate pathway (PPP), the arachidonic acid (AA) metabolic pathway, and amino acid metabolism in RA. These investigations have revealed altered levels of intermediate metabolites in RA compared to healthy controls. For example, RA muscles exhibit elevated pyruvate levels, indicating increased glycolytic activity. Conversely, levels of alpha-ketoglutaric acid and citric acid, intermediates of the TCA cycle, are typically reduced, while succinic acid levels are elevated. Furthermore, disruptions in amino acid metabolism are observed in RA patients, with varying levels of proline, tryptophan, cysteine, and glutamine compared to healthy controls [11, 12].

In this study, we sought to assess the relationship between serum metabolomics and clinical manifestations as well as immunological abnormalities in RA. Our aim was to gain deeper insights into the underlying pathophysiological mechanisms of RA.

## Methods

### Patients and control groups

A total of 35 patients were recruited for this study from Peking University People’s Hospital. Selection criteria for participants included: (1) Diagnosis of RA according to the American College of Rheumatology clinical diagnostic criteria; (2) RA duration exceeding three months; (3) Seropositivity for RA in all patients. A control group comprising 37 healthy volunteers, matched in terms of age and gender, was also included. Individuals with heart disease, diabetes, other underlying disorders, HIV or AIDS, a history of severe smoking and alcohol misuse, or other autoimmune diseases were excluded from participation. All participants provided informed written consent, and the ethics committee approved the study (Ethics Committee Approval Number: 2022PHB345-001).

### Sample collection and preparation

Blood samples were collected from participants using EDTA anticoagulation tubes, with all samples drawn in the early morning in a fasting state. After centrifugation at 3500 rpm for 10 min, the supernatant was collected and stored at -80 °C. All samples used in the study were thawed simultaneously. Upon thawing, 100 μL of each thawed sample was transferred to a 1.5 mL centrifuge tube, followed by the addition of 400 μL of methanol and vortexing for 60 s. As internal standards, 60 μL of 2-Chloro-L-phenylalanine (0.2 mg/mL) and 60 μL of heptadecanoic acid (0.2 mg/mL) were added, followed by another 60 s of vortexing. After centrifugation at 12,000 rpm and 4 °C for 10 min, the supernatant was transferred to another 1.5 mL centrifuge tube. The sample was concentrated using a vacuum centrifuge concentrator, followed by the addition of 60μL of methoxyamine pyridine solution and a 30-s vortexing step. The reaction was allowed to proceed for 120 min at 37 °C. Finally, 60 μL of BSTFA reagent (containing 1% TMCS) was added, and the reaction continued for 90 min. After centrifugation at 12,000 rpm for 10 min, the supernatant was collected in a test vial. For analysis, 20 μL of each sample was combined with a quality control (QC) sample to calibrate the mixed samples, while the remaining samples were subjected to GC–MS analysis.

### GC/MS analysis

The derivatized samples were separated using gas chromatography on an HP-5MS capillary column at a constant flow rate of 1 mL/min with helium as the carrier gas. The autosampler injected 1μL of each sample in split mode with a 20:1 split ratio. The injection temperature was set at 280 °C, the interface temperature at 150 °C, and the ion source temperature at 230 °C. The temperature program consisted of an initial temperature of 60 °C for 2 min, followed by a ramping rate of 10 °C/min up to 300 °C, and a hold at 300 °C for 5 min. Full-scan mass spectrometry was performed within the range of 35 to 750 m/z. To minimize injection order-related discrepancies, all samples were analyzed in a randomized sequence.

### Data processing

The raw data were converted to netCDF format (the input format for XCMS) using G1701 MSD ChemStation software (E.02.00.493). Peak identification, filtration, and alignment were carried out using the XCMS package in R (v3.3.2), generating a data matrix that included information on mass-to-charge ratio (m/z), retention time (rt), and peak intensity. Metabolites with ion peaks missing in more than 50% of samples were excluded from further analysis. Metabolite annotation was performed using the AMDIS software, leveraging the National Institute of Standards and Technology (NIST) commercial database and the Wiley Registry metabolomics database. The annotated data were exported to Excel for subsequent analysis. To ensure comparability across samples with varying intensity scales, the data were normalized using total peak area (SUM normalization).

Before conducting multivariate statistical analysis on metabolomics data, it is typically necessary to apply appropriate scaling methods. In this experiment, data were processed using Pareto scaling (mean-centering and scaling to Pareto variance) prior to multivariate analysis to obtain more reliable and interpretable results. Principal Component Analysis (PCA) and Orthogonal Partial Least Squares Discriminant Analysis (OPLS-DA) were performed using the R package *ropls*.

### Statistical analysis

The study results were presented as mean ± standard deviation (SD). Depending on the normality of variable distributions, either the t-test or non-parametric test of SPSS software version 22.0 was used to calculate p-values, with *p* < 0.05 considered as statistically significant. In this study, Pearson correlation analysis was employed if the quantitative data followed a normal distribution. If the data did not follow a normal distribution or were categorical in nature, Spearman correlation analysis was used instead. Correlation heat maps were generated using the corrplot package within the R platform. All graphs in this study were created using GraphPad Prism version 9.0.

## Results

### Characteristics of participants

Table 1 summarizes the key characteristics of the study participants. The incidence of common attributes such as gender, age, and BMI exhibited no substantial differences between the RA and HC groups (*p* > 0.05).

**Table 1:** Demographics and clinical characteristics of RA patients and healthy controls

Characteristics	RA patients	Healthy controls	*p*-value
Number of samples	35	37	-
Age (mean ± SD)	57 ± 8	56 ± 5	0.476
Gender (F/M)	28 / 7	28 / 9	-
BMI (kg/m^2^)	22.80 ± 3.299	24.26 ± 3.304	0.064
Disease duration (years)	12.60 ± 11.62	-	-
RF (IU/ml)	730.0 (19.00–5840.00)	-	-
ESR (mm/h)	44.74 (5.00–110.00)	-	-
CRP (mg/l)	27.77 (0.35–166.20)	-	-

*RF*, rheumatoid factor; *ESR*, erythrocyte sedimentation rate; *CRP*, C-reactive protein

### Altered metabolic profiles

**Fig. 1:**
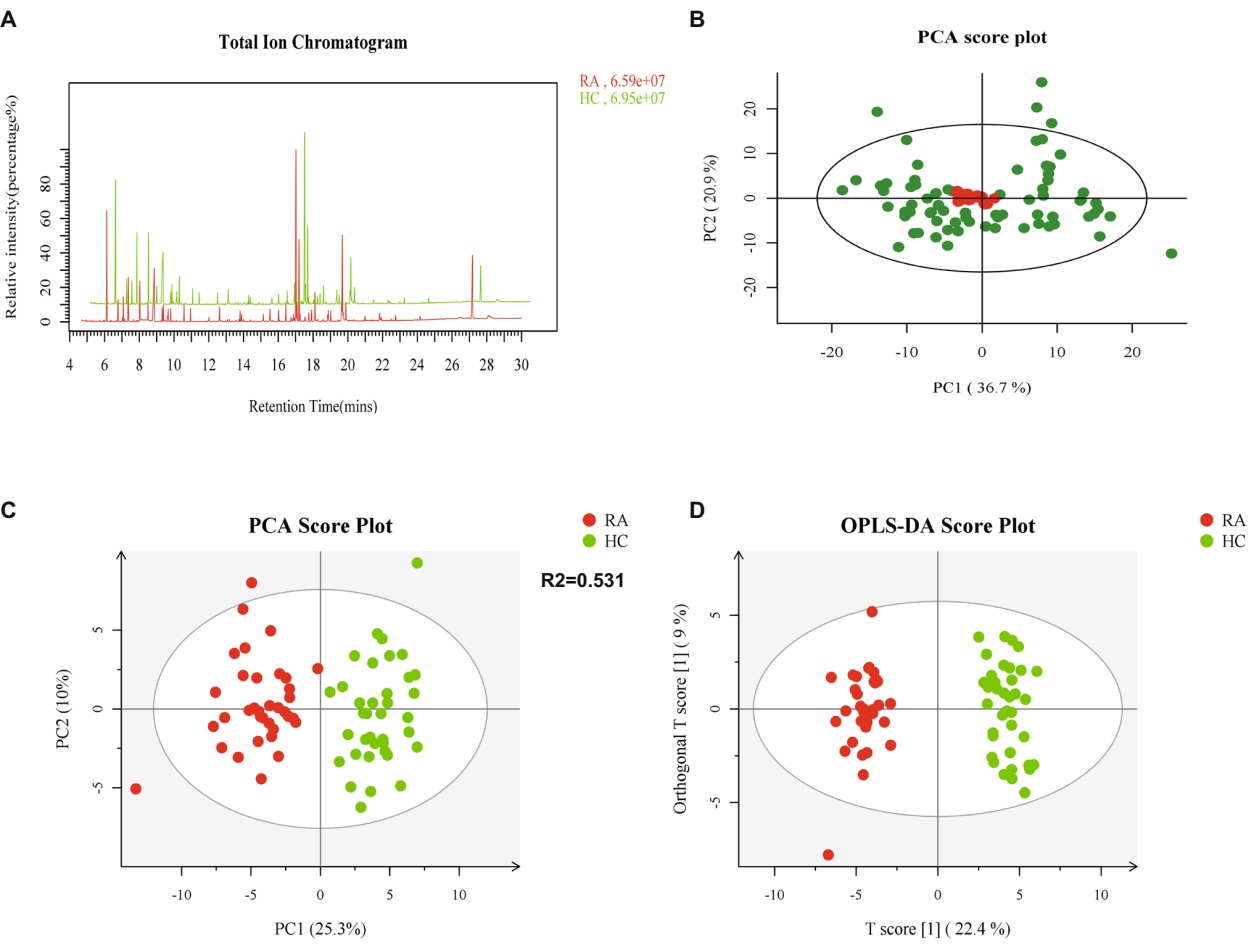
Metabolite principal component analysis, **A** Total Ion Current (TIC) Chromatogram of Serum Samples. **B** PCA Score Plot of QC Samples Showing Method Stability. **C** PCA Score Plot Differentiating RA and HC Groups Based on Serum Metabolites. **D** OPLS-DA Plot Highlighting Inter-group Separation Between RA and HC Groups

The total ion current (TIC) chromatogram (Fig. 1A) was generated by summing the intensities of all ions in each mass spectrum scan and plotting ion intensity against time. In mass spectrometry-based metabolomics studies, quality control (QC) is essential to ensure reliable and high-quality data. The smaller the variation between QC samples, the higher the method's stability and data quality, which is reflected in the PCA plot as a tight clustering of QC samples (Fig. 1B). To visualize the distribution of detected metabolites across serum samples from all participants, we generated a two-dimensional PCA score plot (Fig. 1C), showing a clear separation between the RA and HC groups. RA samples predominantly clustered on the negative side of the plot, while HC samples clustered on the positive side, highlighting the differences in serum metabolite composition between the two groups. Another commonly used method in metabolomics data analysis is Orthogonal Projections to Latent Structures Discriminant Analysis (OPLS-DA), which reduces model complexity and enhances interpretability, allowing for the maximization of group differences (Fig. 1D). The results show clear inter-group separation and minimal intra-group variation. From the OPLS-DA analysis, each metabolite was assigned a Variable Importance in Projection (VIP) score, with metabolites having a VIP value greater than 1 considered as differential metabolites.

**Fig. 2:**
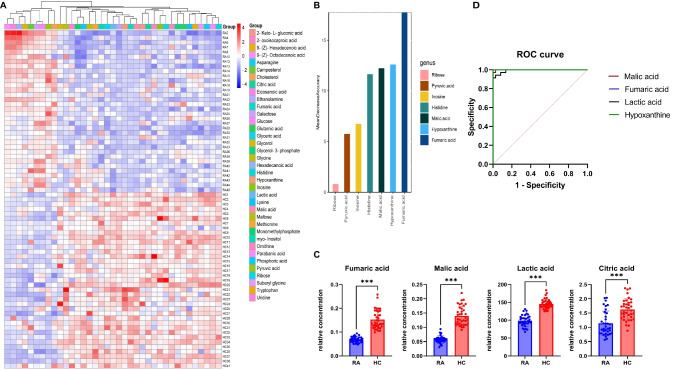
Metabolite variability analysis, **A** Heat map of metabolite differences between RA patients and healthy controls. **B** Average lower abundance of differential metabolites. **C** Significantly different metabolites (malic acid, fumaric acid, citric acid, and lactic acid). **D** Corresponding ROC curves for malic acid, fumaric acid, citric acid, and lactic acid. Data are the mean ± SD.****p < 0.001 by one-way ANOVA

Our analysis identified 37 metabolites that exhibited significant differences between RA and HC (*t*-test, *p* < 0.01), as detailed in Table 2. Among these, nine metabolites, including glucose, galactose, pyruvic acid, eicosanoic acid, hexadecanoic acid, 9-(Z)-Octadecenoic acid, 9-(Z)-Hexadecenoic acid, campesterol, and cholesterol, were enriched in RA, while the rest, such as fumaric acid, malic acid, and citric acid, were depleted in RA (Fig. 2A, C).

**Table 2:** Metabolite concentrations in serum samples

	RA patients	Healthy controls	*p*-value
Ethanolamine	1.067 ± 0.223	1.958 ± 0.262	< 0.001
Malic acid	0.057 ± 0.012	0.140 ± 0.033	< 0.001
Lactic acid	98.54 ± 15.66	145.3 ± 13.55	< 0.001
Fumaric acid	0.068 ± 0.011	0.152 ± 0.039	< 0.001
Parabanic acid	0.098 ± 0.024	0.171 ± 0.027	< 0.001
Histidine	3.273 ± 1.244	6.726 ± 1.237	< 0.001
Hypoxanthine	0.217 ± 0.103	1.850 ± 0.830	< 0.001
Asparagine	0.703 ± 0.12	1.016 ± 1.016	< 0.001
9-(Z)-Octadecenoic acid	41.33 ± 10.99	22.63 ± 3.421	< 0.001
Galactose	193.8 ± 19.59	155.7 ± 16.33	< 0.001
Glyceric acid	0.358 ± 0.069	0.516 ± 0.086	< 0.001
Phosphoric acid	25.74 ± 5.683	34.92 ± 3.829	< 0.001
Glucose	133.9 ± 14.12	109.8 ± 13.90	< 0.001
Tryptophan	6.928 ± 2.101	10.12 ± 1.615	< 0.001
Glutamic acid	9.710 ± 3.075	13.72 ± 1.723	< 0.001
myo-Inositol	2.411 ± 0.522	3.171 ± 0.431	< 0.001
Pyruvic acid	1.042 ± 0.426	0.612 ± 0.727	0.003
Inosine	0.06 ± 0.038	0.203 ± 0.121	< 0.001
Ribose	0.222 ± 0.111	0.733 ± 0.443	< 0.001
Glycerol-3-phosphate	1.307 ± 0.489	2.109 ± 0.545	< 0.001
Eicosanoic acid	0.753 ± 0.177	0.552 ± 0.094	< 0.001
Ornithine	7.125 ± 2.505	10.27 ± 2.021	< 0.001
Monomethylphosphate	1.158 ± 0.259	1.420 ± 0.099	< 0.001
Suberyl glycine	0.393 ± 0.169	0.616 ± 0.173	< 0.001
2-Keto-L-gluconic acid	0.042 ± 0.014	0.060 ± 0.015	< 0.001
Hexadecanoic acid	24.2 ± 5.76	18.5 ± 3.39	< 0.001
Citric acid	1.139 ± 0.43	1.623 ± 0.37	< 0.001
Campesterol	0.119 ± 0.029	0.0828 ± 0.03	< 0.001
2-oxoisocaproic acid	0.079 ± 0.011	0.094 ± 0.013	< 0.001
Methionine	1.625 ± 0.342	2.042 ± 0.357	< 0.001
9-(Z)-Hexadecenoic acid	0.94 ± 0.251	0.638 ± 0.263	< 0.001
Maltose	0.237 ± 0.133	0.43 ± 0.217	< 0.001
Glycerol	5.199 ± 1.119	7.607 ± 2.961	< 0.001
Glycine	16.24 ± 5.170	22.60 ± 6.657	< 0.001
Cholesterol	29.12 ± 4.163	24.78 ± 4.028	< 0.001
Uridine	0.157 ± 0.032	0.191 ± 0.033	< 0.001
Lysine	8.01 ± 1.705	9.938 ± 1.961	< 0.001

Among the 37 differential metabolites, histidine, citric acid, hypoxanthine, and fumaric acid emerged as critical selection targets in the exploratory panel analysis based on mean decrease accuracy, with fumaric acid ranking as the most significant (Fig. 2B).

We further conducted a traditional univariate Receiver Operating Characteristic (ROC) curve analysis for the 37 differential metabolites, constructing ROC curves and calculating sensitivity, specificity, and Area Under the Curve (AUC)for each specific metabolite. As illustrated in Fig. 2D, several metabolites exhibited AUCs surpassing 0.99, including fumaric acid (1.000 (1.000–1.000)), hypoxanthine (1.000 (1.000–1.000)), malic acid (0.999 (0.997–1.000)), and lactic acid (0.993 (0.982–1.000)).

### Metabolic pathways enrichment analysis

**Fig. 3:**
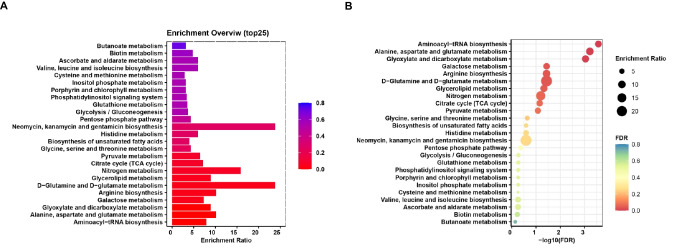
Enrichment of differential metabolic pathways, Top 250 enrichment overview of metabolic pathways shown with (**A**) bar charts (**B**) bubble charts

Simultaneously, we conducted a pathway enrichment analysis on the differential metabolites and visualized the results using bar charts (Fig. 3A) and bubble charts (Fig. 3B). The outcomes indicated that these metabolites were primarily associated with the tricarboxylic acid cycle, glucose metabolism, amino acid metabolism, and fatty acid synthesis. Notably, amino acid biosynthesis exhibited a higher degree of enrichment. Furthermore, pathways related to neomycin, kanamycin, and gentamicin biosynthesis, as well as D-Glutamine and D-glutamate metabolism, displayed an increased number of enriched genes. In comparison to other pathway enrichments, glyoxylate and dicarboxylate metabolism, alanine, aspartate, and glutamate metabolism, along with aminoacyl-tRNA biosynthesis, demonstrated significant levels of enrichment.

### Correlation analysis of differential metabolites with clinical indexes of RA

**Fig. 4:**
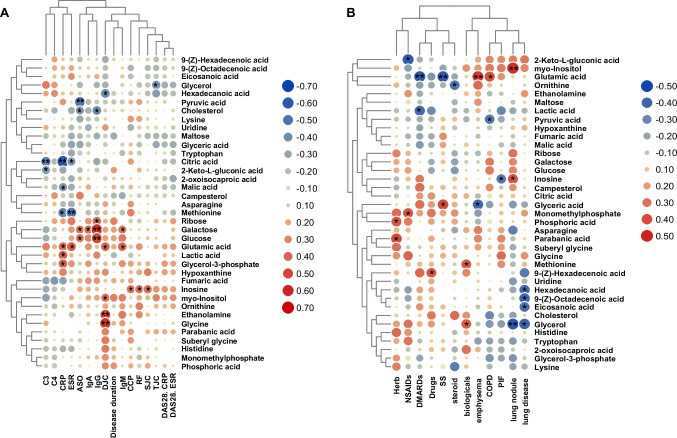
Association analysis between differential metabolites and clinical indicators, Association analysis of differential metabolites with (**A**) common clinical indicators and (**B**) comorbidities and medications. *p < 0.05, **p < 0.01 by one-way ANOVA

The diagnosis of RA involves assessing the type and number of affected joints, serological analysis for rheumatoid factor (RF) and anti-citrullinated protein antibodies (ACPA) concentration, acute phase reactants, and symptom duration, all based on established classification standards. Disease activity and inflammation in RA patients are commonly evaluated using parameters such as C-reactive protein (CRP), erythrocyte sedimentation rate (ESR), Disease Activity Score-28 with CRP (DAS28-CRP), and Disease Activity Score-28 with ESR (DAS28-ESR). Additionally, elevated serum complements C3 and C4 levels have been linked to disease activity in RA. The presence of IgM and IgG antibodies is also relevant to symptom development and prognosis in RA patients. We conducted Spearman correlation tests to examine the relationship between clinical indices and serum metabolites in RA patients (Fig. 4A).

Among the 37 differential metabolites, 22 exhibited a negative correlation with C3, with citric acid demonstrating a significant negative correlation (*R* = -0.445, *p* = 0.007). Similarly, 25 differential metabolites were negatively correlated with C4, and 21 showed negative correlations with CRP, while 15 exhibited positive correlations. Notably, citric acid displayed a significant negative correlation with CRP (*R* = -0.600, *p* = 0.0001), and lactic acid exhibited a positive correlation (*R* = 0.401, *p* = 0.018). We identified 21 metabolites that demonstrated a positive correlation with IgG, with galactose (*R* = 0.567, *p* = 0.0005) and glucose (*R* = 0.492, *p* = 0.003) displaying strong correlations. Additionally, galactose exhibited positive correlations with IgA (*R* = 0.359, *p* = 0.034) and IgM (*R* = 0.344, *p* = 0.043). We also explored correlations between metabolites and swollen joint count (SJC), tender joint count (TJC), and deformity joint count (DJC), revealing a positive correlation between DJC and glycine (*R* = 0.533, *p* = 0.001), as well as ethanolamine (*R* = 0.489, *p* = 0.003). We present the data showing correlations at *p* < 0.05 in full in Supplementary Material Table 1.

We then investigated correlations between different metabolites and drug uptake profiles as well as comorbidities in RA patients, as depicted in Fig. 4B. RA combined with lung nodules exhibited a positive correlation with myo-inositol (*R* = 0.463, *p* = 0.005), while lung nodule development in RA was negatively correlated with glycerol levels (*R* = -0.439, *p* = 0.008). Glutamic acid displayed a negative association with RA comorbid with Sjögren's syndrome (SS) (*R* = -0.434, *p* = 0.009) and disease-modifying antirheumatic drugs (DMARDs) (*R* = -0.433, *p* = 0.009), but exhibited a positive correlation with RA combined with emphysema (*R* = 0.434, *p* = 0.009). We present the data showing correlations at *p* < 0.05 in full in Supplementary Material Table 2.

### Analysis of the correlation between differential metabolites and immune cells in RA

**Fig. 5:**
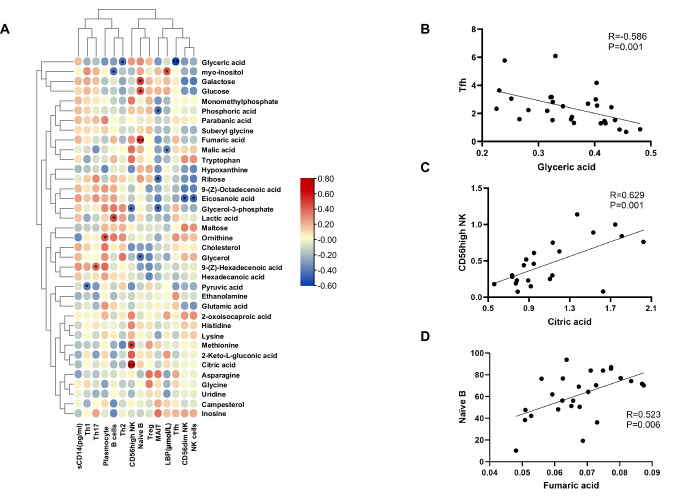
Association analysis of differential metabolites with cellular subpopulations, Analysis of differential metabolites associated with (**A**) cellular subpopulations and (**B**-**D**) between metabolites with significant associations. *p < 0.05, **p < 0.01 by one-way ANOVA

Given the observed immune cell imbalance in RA patients, we collected peripheral blood samples and used flow cytometry to identify immune cells and record their proportional content. Subsequently, we applied Spearman correlation testing to investigate potential associations between immune cells and blood metabolites (Fig. 5A). We assessed the proportions of Th1(T helper cell 1), Th2(T helper cell 2), Th17(T helper cell 17), Treg(Regulatory T cell), and Tfh(T follicular helper cell) cells in CD4 + cells and analyzed their correlation with metabolic products. We identified a negative correlation between glyceric acid and Tfh cells (Fig. 5B). We further examined NK cells, whose role is mainly influenced by the NK subset identified by CD56, and their correlation with metabolites. Specifically, we distinguished between highly cytotoxic CD56^dim^ NK cells, which provide efficient clearance of malignant or virus-infected cells through either cell-dependent or antibody-dependent cytotoxicity, and CD56^bright^ NK cells, which produce multiple cytokines and significantly contribute to the regulation of immune response and maintenance of homeostasis. This analysis revealed a positive association between citric acid and CD56^high^ NK cells (Fig. 5C). In the case of B lymphocytes, encompassing naive B cells and plasma cells, we explored correlations with metabolites and identified a positive correlation between fumaric acid and naive B cells (Fig. 5D). We present the data showing correlations at *p* < 0.05 in full in Supplementary Material Table 3.

## Discussion

Metabolomics has become an invaluable tool in the study of autoimmune disorders, particularly in the identification of disease-predicting biomarkers based on alterations in metabolite profiles. Previous investigations have uncovered metabolic disparities between HC and RA patients by analyzing serum, urine, and synovial fluid samples [8]. Consistent with these earlier studies, we observed reductions in metabolites such as citric acid [12], histidine [13], and glutamate [14], along with increases in glucose and galactose [15] metabolism. However, in contrast to some studies [16], we also identified a reduction in fumaric acid levels. These metabolic changes are intricately linked to various biological reactions, including those associated with the TCA cycle, glycolysis, lipid metabolism, and amino acid metabolism.

Notably, RA patients displayed significantly lower amino acid levels but elevated concentrations of glucose and lactate. Apart from their role in providing energy for physical activities, glucose metabolism also orchestrates a signaling network that coordinates diverse physiological processes. The reduced amino acid levels in RA suggest that protein breakdown into amino acids is required to maintain energy homeostasis, mitigate inflammation, and support autoimmunity [15]. The downregulation of citric acid and fumaric acid in RA patients signifies a dampened aerobic metabolic process. The citric acid cycle, a pivotal component of aerobic catabolism, experiences diminished citric and fumaric acid levels, indicative of reduced energy production during inflammation [17]. Several biological reactions implicated in RA, including glutamine catabolism, glycolysis, amino acid and fatty acid synthesis, and choline metabolism, serve as potential therapeutic targets [18–21]. Citric acid's close association with Alzheimer's disease activity suggests its potential as a diagnostic metabolite for the condition [22]. Given the observed differences in fumaric acid levels between RA and HC, it may hold promise as a diagnostic marker in the future.

RA is characterized by dysregulated immune responses and is predominantly driven by CD4 + T lymphocytes, with contributions from IFN-producing Th1 cells and IL-17-producing helper T cells (Th17) [23]. Additionally, it is well-known that pro-inflammatory mediators can induce expansion of regulatory T cells (Treg), which was observed in RA joints [24]. B cells also play a significant role in RA pathophysiology, influencing antigen presentation, autoantibody production, and cytokine release, thus making them integral to the disease's development. Emerging evidence suggests that Tph and Tfh cells may be more abundant in RA patients compared to HC [25]. Glycolic acid, a key component of glycolysis, exhibited reduced levels in RA patients and displayed a negative correlation with Tfh cells, suggesting a potential link to Tfh cell differentiation and proliferation, although further evidence is needed to substantiate this connection. NK cells, critical regulators of the immune response, exhibit distinct subsets characterized by CD56 expression [26]. Citric acid and fumaric acid, both involved in the TCA cycle and possessing antioxidant properties, exhibited positive correlations with NK cells and naive B cells.

In this study, we employed univariate and multivariate statistical analyses to identify 37 differential metabolites in RA, shedding light on their potential association with disease activity and immune dysregulation. Nevertheless, our research possesses certain limitations, including a limited sample size and the absence of validation studies. To validate the hypotheses generated in this study, additional experimental studies involving larger, independent sample cohorts are warranted.

## Conclusion

In conclusion, significant differences exist in serum metabolic profiles between RA patients and healthy individuals. These altered metabolites are intricately linked to disease activity and immune dysregulation. Ultimately, our findings contribute to a deeper understanding of the underlying pathophysiological mechanisms of RA.

## Supplementary Information

Below is the link to the electronic supplementary material.

Supplementary file1 (XLSX 15 KB)

## Data Availability

The data that support the findings of this study are available from the corresponding author, upon reasonable request.
